# Oxidative stress induces imbalance of adipogenic/osteoblastic lineage commitment in mesenchymal stem cells through decreasing SIRT1 functions

**DOI:** 10.1111/jcmm.13356

**Published:** 2017-10-03

**Authors:** Chia‐Hua Lin, Nan‐Ting Li, Hui‐Shan Cheng, Men‐Luh Yen

**Affiliations:** ^1^ Department of Obstetrics/Gynecology National Taiwan University (NTU) Hospital & College of Medicine NTU Taipei Taiwan

**Keywords:** mesenchymal stem cells, reactive oxygen species, adipogenesis, osteogenesis, SIRT1, FOXO3a

## Abstract

With rapidly ageing populations worldwide, the incidence of osteoporosis has reached epidemic proportions. Reactive oxygen species (ROS), a by‐product of oxidative stress and ageing, has been thought to induce osteoporosis by inhibiting osteogenic differentiation of mesenchymal stem cells (MSCs). However, specific mechanisms of how ROS results in alterations on MSC differentiation capacity have been inconsistently reported. We found that H_2_O_2_, an ROS, simultaneously induced MSC lineage commitment towards adipogenesis and away from osteogenesis at the functional as well as transcriptional level. In addition, H_2_O_2_ decreased the activities of SIRT1, a histone deacetylase and longevity gene. By silencing and reconstituting SIRT1 in MSCs, we demonstrated that H_2_O_2_ exerted its disparate effects on adipogenic/osteoblastic lineage commitment mainly through modulating SIRT1 expression levels. Treatment with resveratrol, a SIRT1 agonist, can also reverse this ROS‐induced adipogenesis/osteogenesis lineage imbalance. Moreover, SIRT1 regulation of RUNX2 transcriptional activity was mediated through deacetylation of the ROS‐sensitive transcription factor FOXO3a. Taken together, our data implicate SIRT1 as playing a vital role in ROS‐directed lineage commitment of MSCs by modulating two lineages simultaneously. Our findings on the critical role of SIRT1 in ROS/age‐related perturbations of MSC differentiation capacity highlight this molecule as a target for maintenance of MSC stemness as well as a potential anabolic target in osteoporosis.

## Introduction

Osteoporosis is a common metabolic disease of bone tissue, which is characterized by low bone mineral density with subsequent increased fracture risk. Both loss of oestrogen in women at menopause and ageing in general are risk factors for this disease 1, 2. Oxidative stress, which is the outcome of excessive reactive oxygen species (ROS) production and/or declining antioxidant activities, is a known driving force in accelerating age‐related changes, including osteoporosis 3. ROS include hydrogen peroxide (H_2_O_2_), superoxide (O_2_^−^) and hydroxyl radical (OH^−^), and at low levels, it can function as secondary messengers and regulate various physiological cellular responses 4; however, excessive ROS can trigger damage in all biomolecules, including DNA/RNA, proteins and lipids 5, 6. Importantly, oxidative stress has been reported to contribute to osteoporosis by decreasing the osteogenic potential of mesenchymal stem cells (MSCs) 7, 8, the progenitors of osteoblasts, adipocytes and chondrocytes.

Stem cell commitment into specific somatic phenotypes is controlled by master lineage transcription factors, and for MSC lineage commitment, the transcription factors PPARγ2, RUNX2 and SOX9 control adipogenesis, osteogenesis and chondrogenesis, respectively 9, 10, 11. In particular, adipogenesis and osteogenesis appear to be mutually exclusive, and antagonism between adipogenic and osteogenic factors has been shown through functional assays: PPARγ2 insufficiency can result in increased osteogenesis through osteoblast formation from bone marrow progenitors 12, 13, whereas RUNX2 depletion can promote adipogenesis 14. This ‘seesaw’ relationship between osteogenesis and adipogenesis in MSCs appears to be particularly prominent in senescence, in which ROS are known to be increased 15, 16, 17. Surprisingly, the detail molecular mechanisms of how oxidative stress directs lineage commitment of MSCs remain largely unexplored.

SIRT1, a histone deacetylase as well as a longevity gene 18, is known to inhibit adipogenesis through indirectly repressing PPARγ2 by binding the nuclear receptor corepressors (NCoR) and silencing mediator of retinoid and thyroid hormone receptors (SMRT) 19. This in turn promotes osteogenesis through activation of RUNX2‐dependent gene transcription 20, 21. Interestingly, SIRT1 itself is a vital antioxidant that regulates transcription and DNA damage repair in response to oxidative stress, and the osteogenic function of SIRT1 requires FOXO3a, a key transcription factor in cellular antioxidant responses 22. We therefore have been suggested that SIRT1 involves in oxidative stress‐induced adipogenesis–osteogenesis lineage switching in MSCs, and that targeting this molecule may represent an anabolic therapeutic option for osteoporosis. Our data demonstrate that exogenous ROS treatment does promote adipogenesis at the expense of osteogenesis in MSCs, and SIRT1 agonism through resveratrol or reconstitution can reverse these effects through deacetylation of FOXO3a.

## Materials and methods

### Cell culture

The mouse MSC cell line C3H10T1/2 was obtained from the American Type Culture Collection (ATCC, Manassas, VA, USA) and cultured as per manufacturer's instructions. Cells were grown in complete medium consisting of BME‐low glucose (Gibco‐Invitrogen, Carlsbad, CA, USA), 100 U/ml penicillin/streptomycin (Gibco‐Invitrogen) and 10% foetal bovine serum (Hyclone, Logan, UT, USA) in a humidified atmosphere of 95% air and 5% CO2 at 37°C. H_2_O_2_ and *N*‐acetyl‐l‐cysteine (NAC) were obtained from Sigma‐Aldrich (St. Louis, MO, USA).

### Differentiation assays and cytochemical staining

Differentiation assays and cytochemical staining for adipogenesis and osteogenesis were performed as our previous report 20, 23. Briefly, for adipogenic differentiation, cells were cultured in complete medium with 10% rabbit serum, 0.5 mM isobutylmethylxanthine, 1 μM dexamethasone and 10 μM insulin (all from Sigma‐Aldrich). Thereafter, cells were fixed with 4% paraformaldehyde and stained with Oil red O solution (Sigma‐Aldrich) for 10 min. After repeatedly washing with 85% propylene glycol and distilled water for 5 min., lipid vacuoles were visualized. For osteogenic differentiation, cells were cultured in complete medium with 0.2 mM l‐ascorbic acid 2‐phosphate, 1 μM dexamethasone and 0.05 mM β‐glycero‐phosphate and replaced every 3 days. Alizarin red staining is performed to analyse calcium deposits. Briefly, cells were fixed with 100% methanol for 30 min., washed with boric acid buffer (0.1 M, pH 4.0) and stained with Alizarin red solution (Sigma‐Aldrich) 40 mM, pH 4.2 for 30 min. After repeated washing with boric acid buffer and distilled water, calcium deposits were seen and visualized. Elution of stains was performed, and absorbance was read at 520 nm.

### Alkaline phosphatase (ALP) activity

Cellular ALP activity was measured by colorimetric assay as previously performed 20. Briefly, protein lysates were incubated with the substrate p‐NPP (Sigma‐Aldrich) at room temperature for 5 hrs. The yellow‐coloured product was measured by reading the absorbance at 405 nm and normalized against the corresponding protein concentration, which was determined by Bradford protein assay (BioRad, Hercules, CA, USA).

### Nile red staining

Flow cytometric quantification of oil droplet formation was performed with Nile red staining. Cultured cells were trypsinized and fixed with 4% paraformaldehyde for 1 hr at 4°C and then subjected to Nile red staining (10 μg/ml) for 45 min. at room temperature. Fluorescence intensity was measured by emission between 564 and 604 nm (FL‐2 channel) by FACScan analyses (BD Biosciences, San Jose, CA, USA).

### Quantitative PCR (qPCR)

RNA extraction was performed as described previously 20. For quantitative PCR (qPCR) assay, each cDNA was amplified using SYBR Green on the ABI Real‐time PCR 7500 System according to the manufacturer's instructions (Applied Biosystems Inc., Carlsbad, CA, USA). Primer sequences were designed using the online Primer3 software (Whitehead Institute for Biomedical Research, Cambridge, MA, USA); the primer sequences for LEPTIN were based on a previous report 24. All primer sequences are listed in Table 1.

**Table 1:** List of primers used for quantitative PCR

Gene symbol	Target gene	Primer sequence (F, forward; R, reverse)	Assay ID	Amplicon length
RUNX2	runt‐related transcription factor 2	F: CCCAGGCGTATTTCAGATGAT R: GGTGTAGGTAAAGGTGGCTG	NM_001146038.2	198
COL1A1	collagen type I alpha 1 chain	F: ATGTTCAGCTTTGCGGACCTC R: CACGTCATCGCACACAGCC	NM_007742.4	192
KLF5	Kruppel‐like factor 5	F: AGGACTCATACGGGCGAGAA R: ATGCACTGGAACGGCTTGG	NM_009769.4	107
KLF2	Kruppel‐like factor 2	F: TGCCGTCCTTTGCCACTTTC R: CCCAGACCGTCCAATCCCAT	NM_008452.2	145
LEPTIN	leptin	F: GAGACCCCTGTGTCGGTTC R:CTGCGTGTGTGAAATGTCATTG	NM_008493.3	139
C/EBPβ	CCAAT/enhancer binding protein, beta	F: CCAACTTCTACTACGAGCC R: AAGAGGTCGGAGAGGAAG	NM_001287738.1	199
TBP	TATA‐box binding protein	F: CAACAACAGCAGGCAGTAGCA R: TGGTGTGGCAGGAGTGATAGG	NM_013684.3	195
PPARγ2	Peroxisome proliferator‐activated receptor gamma 2	F: GGGTGAAACTCTGGGAGATTCT R: CTGTGGTAAAGGGCTTGATGTC	NM_011146.3	200
c‐MAF	Mus musculus avian musculoaponeurotic fibrosarcoma oncogene homolog	F: CACTTCGACGACCGCTTCT R: GTCCGCCTCTTCTGCTTCA	NM_001025577.2	131

### Plasmids, transient transfection and promoter–luciferase reporter assay

The information of human RUNX2 promoter–luciferase reporter (RUNX2‐Luc) was described in our previous publication 20. The plasmids, including Flag‐SIRT1 22, Flag‐SIRT1 H363Y 22, HA‐FOXO3a 25 and FHRE‐Luc 25, were purchased from Addgene (Cambridge, MA, USA). Cells were transfected using the Lipofectamine 3000 reagent according to the manufacturer's instructions (Life Technologies, Carlsbad, CA, USA). After 24 hrs, the medium of transfected cells was changed into fresh complete medium with various treatments. Luciferase activity was measured using the Promega Luciferase Assay System and standardized against β‐galactosidase activity (Promega, Madison, WI, USA). Values were shown as the mean (± S.D.) of three replicates and at least three independent trials.

### Western blotting and antibodies

Western blotting analyses were performed as described previously 23, with the following primary antibodies used: β‐actin and α‐tubulin were obtained from Sigma‐Aldrich; SIRT1 was purchased from Santa Cruz Biotechnology (Santa Cruz, CA, USA); and FOXO3a and acetylated lysine (Acetyl‐K) were purchased from Cell Signaling Technology (Danvers, MA, USA).

### Immunoprecipitation (IP)

Protein lysates (500 μg) were pre‐incubated with the antibody of interest (1 μg) for 2 hrs on a rotator at 4°C. Next, 20 μl of PureProteome Protein A magnetic beads (Millipore, St Charles, MO, USA) was added and incubated for additional 1 hr at 4°C. The immunoprecipitated complex was pulled down by the magnetic rack, washed with PBS/0.1% Tween 20 surfactant three times and eluted into 40‐μl electrophoresis buffer by heating at 90°C for 10 min. Western blotting was performed to analyse the components of IP complex.

### ROS measurement

Intracellular ROS were detected by the peroxide‐sensitive fluorophore 2′,7′‐dichlorodihydrofluorescein diacetate (DCF‐DA) (Gibco‐Invitrogen). Cells were incubated with 10 μM of DCF‐DA for 1 hr at 37°C and then washed with PBS. Suspended cells were analysed by emission with 488 nm by FACScan flow cytometry (BD Biosciences).

### Statistical analyses

All experimental results and measurements are triplicates and expressed as the mean ± standard deviation (S.D.). To confirm reproducibility, all experiments were repeated at least three times. Statistical analyses were performed with Student's *t‐*test.

## Results

### H_2_O_2_‐induced oxidative stress favours adipogenesis at the expense of osteogenesis in MSCs

**Figure 1:**
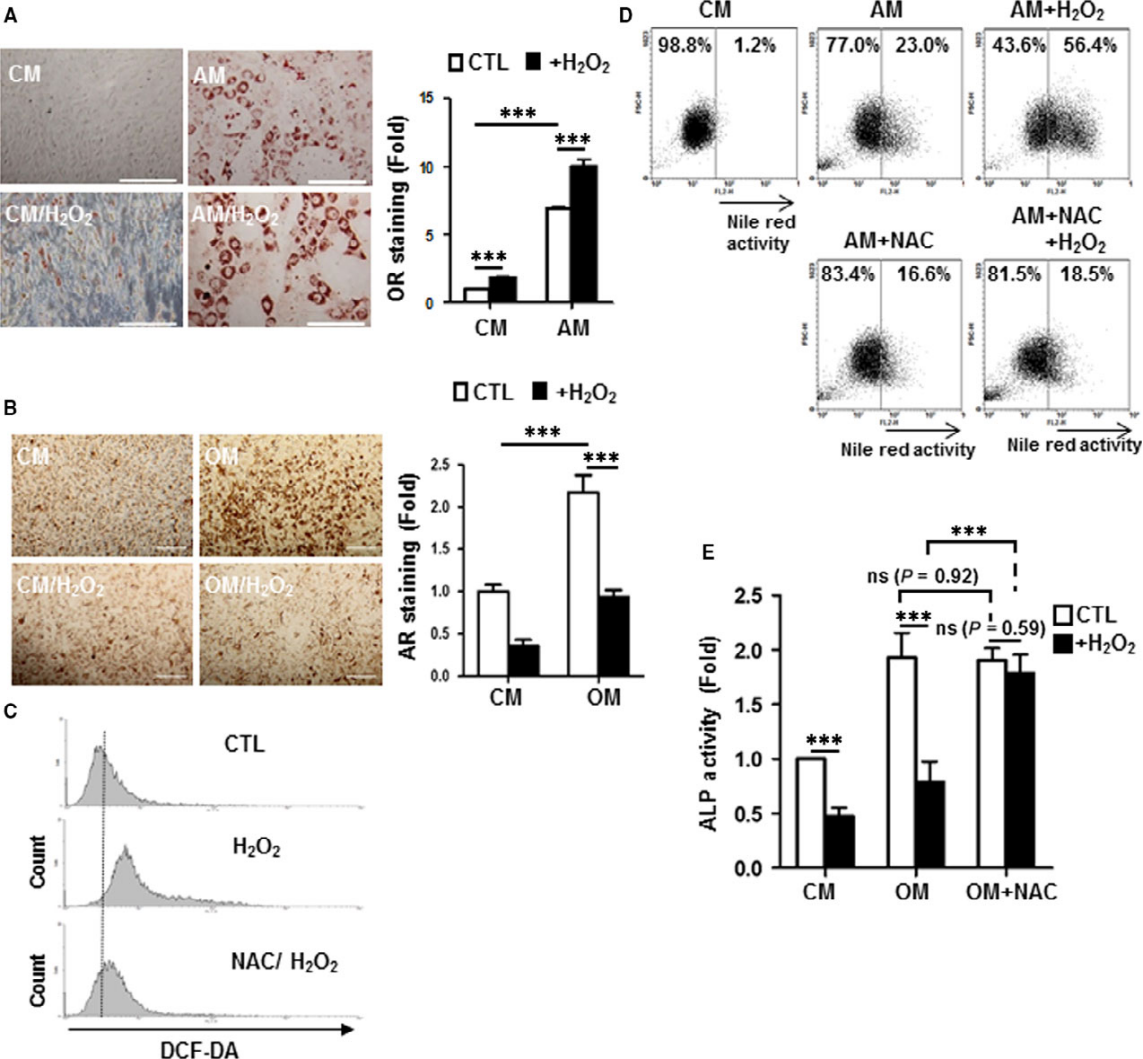
H_2_O_2_‐induced reactive oxygen species (ROS) stress increases adipogenic but decreases osteogenic differentiation capacity of mesenchymal stem cells (MSCs). Effects of H_2_O_2_ on (**A**) adipogenic and (**B**) osteogenic differentiation of C3H10T1/2 (C3H) MSCs. For adipogenic differentiation, cells were cultured in complete medium (CM) or adipogenic medium (AM) for 3 days without and with addition of H_2_O_2_ (30 μM) as evaluated by Oil Red O staining for oil droplet formation. For osteogenic differentiation, cells were cultured in CM or osteogenic medium (OM) for 7 days without and with the presence of H_2_O_2_ as analysed by Alizarin red staining for calcium deposition. The stained cells were photographed (left panel) and then quantified by elution of stains with subsequent spectrophotometric analyses (right panel). (**C**) Intracellular ROS quantification in C3H MSCs treated with H_2_O_2_ without or with the addition of *N*‐acetyl‐l‐cysteine (NAC; 10 mM) for 24 hrs as assessed by 2′,7′‐dichlorodihydrofluorescein diacetate (DCF‐DA) staining and subsequent FACScan analyses. (**D**) Quantification of adipogenic differentiation in C3H MSCs cultured in AM for 3 days in the presence of H_2_O_2_ or NAC as assessed by Nile red staining with subsequent FACScan flow cytometry analyses for oil droplet deposition. (**E**) Quantification of osteogenic differentiation in C3H MSCs cultured in OM for 7 days in the presence of H_2_O_2_ or NAC as measured by alkaline phosphatase (ALP) activity. All quantitative data are shown as fold‐change relative to control conditions (in CM); ***, *P* < 0.001; ns, non‐significant.

To elucidate the molecular mechanisms involved in ROS modulation of MSC lineage commitment, we first assessed the functional effects of ROS on MSC lineage commitment using a mouse MSC line C3H10T1/2 (C3H) 26, 27. As ROS is well known to affect cell proliferation and viability, we first established the dose–response effect of H_2_O_2_ on C3H viability, which we determined to be 30 μM. This sublethal H_2_O_2_ concentration was subsequently used in all MSC differentiation experiments. We found that H_2_O_2_ treatment further enhanced adipogenesis of C3H MSCs cultured in adipogenic induction medium (AM) by 1.4‐fold as demonstrated by Oil Red O staining for oil droplet formation. Surprisingly, the enhancement of adipogenesis was even more apparent when H_2_O_2_ was added to C3H cultured in basal medium (control medium; CM), with a 1.8‐fold increase in oil droplet formation (Fig. 1A). In contrast, while C3H cultured in osteogenic induction medium (OM) demonstrated a twofold increase in calcium deposition over culturing in CM, as measured by Alizarin red staining, this was completely abolished when H_2_O_2_ was added (Fig. 1B). Our data therefore demonstrate that exogenous H_2_O_2_ enhanced adipogenic commitment while diminishing osteogenic commitment in MSCs; moreover, H_2_O_2_ alone can induce MSC adipogenesis and abolish osteogenesis.

ROS scavengers such as *N*‐acetyl‐l‐cysteine (NAC) can reduce ROS levels, and to determine whether ROS‐mediated alteration of MSC differentiation capacity can be reversed with scavenging of ROS, we first assessed whether intracellular ROS levels in MSCs were affected with exogenously added H_2_O_2_. We found that this indeed resulted in increases in intracellular ROS levels, which can be reversed with the ROS scavenger NAC (Fig. 1C). To assess whether scavenging of ROS can reverse the alteration in MSC differentiation capacity brought upon with exogenously added H_2_O_2_, we added NAC to H_2_O_2_‐treated C3H MSCs cultured in CM, AM or OM. H_2_O_2_ treatment increased oil droplet formation from 23% (AM‐only) to 56.4% (AM/H_2_O_2_) as assessed by Nile red staining with flow cytometric analyses (Fig. 1D); however, addition of NAC not only reversed the adipogenic effects of exogenous H_2_O_2_ (18.5%), but also could neutralize the constitutive adipogenic induction of AM‐only as well (16.6%). On the other hand, addition of NAC reversed the inhibitory effect of H_2_O_2_ on osteogenesis as measured by alkaline phosphatase (ALP) activity assay, an early osteogenic marker 28 (Fig. 1E). Interestingly, NAC addition reduced adipogenesis under normal adipogenic differentiation condition (Fig. 1D) but did not affect ALP activity when H_2_O_2_ was not added under normal osteogenic differentiation condition (Fig. 1E), suggesting that endogenous ROS were generated during adipogenic process. Taken together, these findings support that ROS scavenging can reverse H_2_O_2_‐induced MSC adipogenic/osteogenic lineage commitment imbalance.

### H_2_O_2_ enhances the transcriptional programme of adipogenesis while suppressing the transcriptional programme of osteogenesis

**Figure 2:**
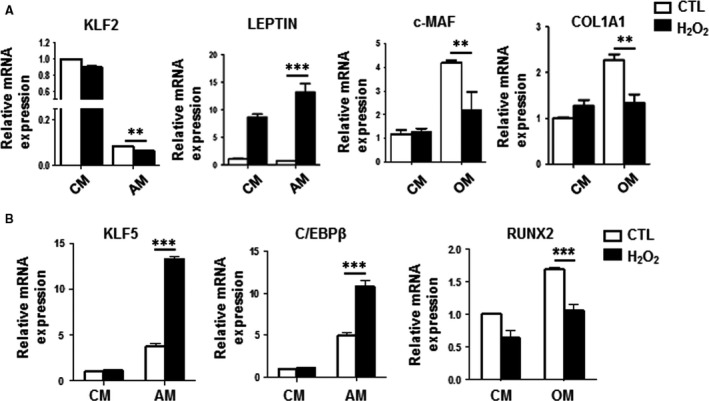
H_2_O_2_ enhances expression of adipogenic‐related genes but inhibits expression of osteoblast‐related genes in mesenchymal stem cells (MSCs). Quantitative PCR (qPCR) analyses of (**A**) downstream adipogenic‐related and osteogenic‐related gene expression levels and (**B**) lineage‐specific transcription factor expression levels in C3H MSCs treated without (CTL) and with H_2_O_2_ under CM, AM or OM for 2 days. **, *P* < 0.01; ***, *P* < 0.001.

We then assessed the effects of H_2_O_2_ on MSC transcriptional programmes of adipogenesis and osteogenesis. Gene expression analyses of lineage‐committed genes in C3H MSCs demonstrated that H_2_O_2_ treatment increased mRNA expression of LEPTIN, a late marker of adipogenic differentiation 29, while expression of KLF2, a repressor of PPARγ2 and adipogenesis 30, 31, was strongly decreased under adipogenic induction at day 2. Moreover, KLF2 expression was further decreased in AM conditions when exogenous H_2_O_2_ was added (Fig. 2A). Conversely, expression levels of committed osteogenic genes such as collagen type 1α1 (COL1A1) 32 and the RUNX2 coactivator c‐MAF 17, 33 were decreased in C3H MSCs cultured in OM with exogenous H_2_O_2_ treatment (Fig. 2A). To ascertain whether master lineage transcription factors were involved, we detected for gene expression of CCAAT‐enhancer‐binding protein β (CEBPβ) and KLF5, which are upstream activators of PPARγ2 to represent early events in adipogenic commitment 31, 34 and RUNX2 to represent early events in osteogenic commitment 32. As shown in Figure 2B, H_2_O_2_ treatment enhanced CEBPβ and KLF5 gene expression levels under AM conditions (Fig. 2B). On the other hand, levels of RUNX2 were significantly down‐regulated by H_2_O_2_ under OM conditions (Fig. 2B). These findings support that H_2_O_2_ treatment enhances the transcriptional programme of adipogenesis while suppressing the transcriptional programme of osteogenesis in MSCs.

### SIRT1 is affected by oxidative stress and can modulate the transcriptional machinery of MSC adipogenesis/osteogenesis

**Figure 3:**
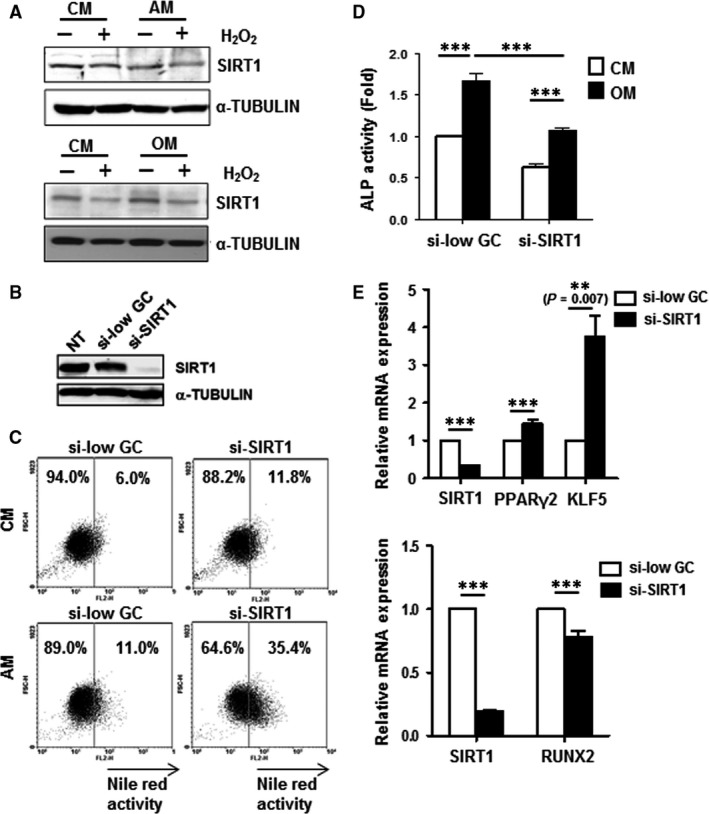
Involvement of SIRT1 in fine‐tuning reactive oxygen species (ROS)‐altered differentiation switch between adipogenic and osteogenic lineage commitment in mesenchymal stem cells (MSCs). (**A**) Effects of H_2_O_2_ on SIRT1 protein expression in C3H MSCs cultured in CM, AM or OM were analysed by Western blotting; α‐TUBULIN, internal control. (**B**) Western blotting verification of SIRT1 knockdown with small interfering RNA (siRNA) in C3H MSCs; non‐target siRNA knockdown (si‐low GC; control) compared with siRNA SIRT1 knockdown (si‐SIRT1). NT, non‐transfected cells (**C**) Adipogenic differentiation capacity of si‐SIRT1‐ and si‐low GC‐C3H MSCs cultured in CM or AM for 2 days, with oil droplet formation assessed and quantified by Nile red staining. (**D**) Osteogenic differentiation capacity of si‐SIRT1‐ and si‐low GC‐C3H MSCs cultured in CM or OM for 6 days as analysed by ALP activity assay. (**E**) qPCR analyses for gene expression levels of KLF5, PPARγ2, RUNX2 and SIRT1 in si‐SIRT1‐ and si‐low GC‐C3H MSCs under differentiation medium. ***, *P* < 0.001.

The deacetylase and longevity gene SIRT1 have been shown independently in numerous reports to enhance osteogenesis and suppress adipogenesis, but whether such functions occur simultaneously and the molecular mechanisms involved have not been clearly demonstrated. In addition, ROS also can modulate SIRT1 function and vice versa 35. To elucidate the molecular mechanisms involved in SIRT1 actions on MSC lineage commitment with and without oxidative stress, we first assessed the expression profile of SIRT1 under H_2_O_2_ treatment together with adipogenic or osteogenic stimulation. We found that in C3H MSCs, culturing in AM reduced endogenous SIRT1 protein levels whereas culturing in OM increased protein levels (Fig. 3A). Importantly, H_2_O_2_ decreased SIRT1 protein expression levels regardless of the differentiation conditions used (Fig. 3A). To evaluate the contribution of H_2_O_2_‐reduced SIRT1 expression on directing MSC lineage commitment towards adipogenesis and/or osteogenesis, knockdown of SIRT1 expression with siRNA in C3H MSCs was performed (Fig. 3B). Knockdown of SIRT1 in C3H MSCs strongly enhanced adipogenic commitment regardless of culturing in CM or AM as measured by Nile red staining for oil droplet formation (Fig. 3C), while osteogenic commitment was decreased regardless of culturing in CM or OM as measured by ALP activity (Fig. 3D). To assess whether adipogenic and osteogenic transcriptional programmes were affected by knockdown of SIRT1 in C3H MSCs, we assessed for gene expression levels of KLF5, PPARγ2 and RUNX2, respectively. We found that knockdown of SIRT1 increased expression levels of KLF5 and PPARγ2 while decreasing levels of RUNX2 (Fig. 3E). Collectively, these findings demonstrate that oxidative stress reduces SIRT1 levels, and that SIRT1 modulates MSC lineage commitment by suppressing adipogenesis and enhancing osteogenesis at the level of master lineage transcription factors.

### Reconstitution of SIRT1 restores oxidative stress‐induced MSC adipogenesis/osteogenesis lineage commitment imbalance

**Figure 4:**
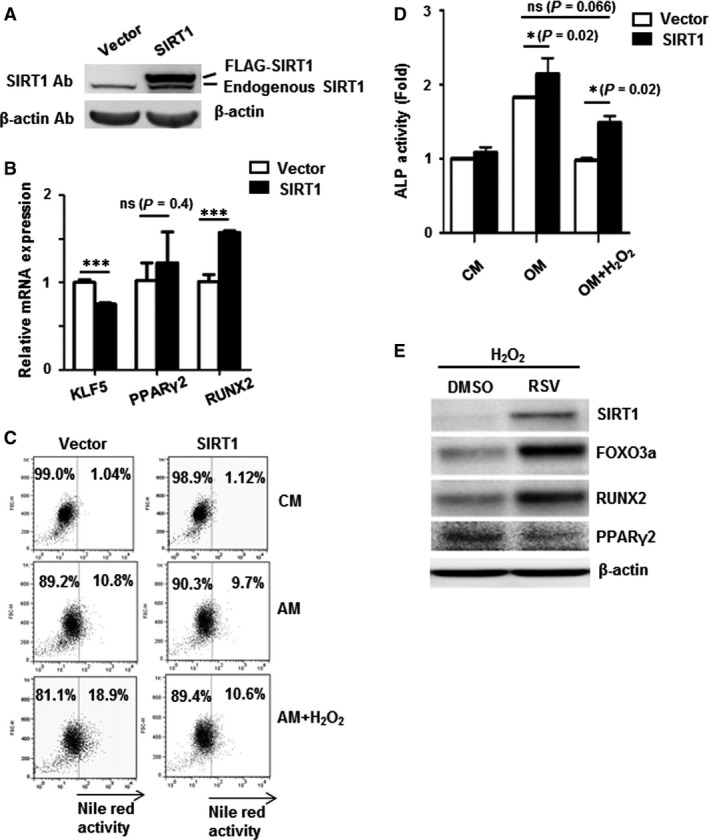
Restoration of SIRT1 expression reverses H_2_O_2_‐induced imbalance in lineage commitment of mesenchymal stem cells (MSCs). (**A**) Western blotting verification on reconstitution of SIRT1 proteins. (**B**) qPCR analyses for gene expression levels of KLF5, PPARγ2 and RUNX2 in SIRT1‐overexpressing cells (**C**) Adipogenic differentiation capacity of C3H MSCs with overexpression of wild‐type SIRT1 (compared to vector overexpression) cultured in AM for 3 days without and with H_2_O_2_ treatment as assessed by Nile red staining for oil droplet formation. (**D**) Osteogenic differentiation capacity of C3H MSCs with overexpression of wild‐type SIRT1 (compared to vector overexpression) cultured in OM for 6 days without and with H_2_O_2_ treatment as assessed by ALP activity. (**E**) Effects of SIRT1 on restoration of reactive oxygen species (ROS)‐induced defects with addition of resveratrol (RSV), a SIRT1 agonist, to H_2_O_2_‐treated cells for 2 days. Western blotting analyses were performed to detect protein expression using the indicated antibodies. β‐actin was used as loading control. *, *P* < 0.05; ns, non‐significant.

To ascertain whether modulation of SIRT1 can restore ROS‐induced MSC adipogenesis/osteogenesis imbalance, we reconstituted SIRT1 expression in H_2_O_2_‐treated C3H MSCs and assessed differentiation capacity. The protein expression of exogenous SIRT1 was verified in Figure 4A. Notably, the reconstitution of SIRT1 could reduce KLF5 gene expression while enhancing RUNX2 gene expression. However, PPARγ2, a downstream gene of KLF5, was not affected at this stage (Fig. 4B). Using the SIRT1 agonist resveratrol (RSV) under H_2_O_2_ stimulation to further induce endogenous SIRT1 expression, we found that RSV could reverse the effects of H_2_O_2_ on inhibition of SIRT1 and FOXO3a protein expression. Moreover, RSV‐induced SIRT1 agonism reversed the H_2_O_2_‐induced decreases in RUNX2 protein levels while reducing the increases in PPARγ2 protein levels brought about by H_2_O_2_ (Fig. 4E). These results indicate that SIRT1 has a vital role in regulating ROS‐induced differentiation transcriptional activity in MSCs. Functionally, Nile red staining showed that oil droplet formation was decreased in SIRT1‐overexpression MSCs (10.6%) in comparison with that in vector‐transfected MSCs (18.9%) cultured in under AM with H_2_O_2_ treatment (Fig. 4C). In contrast, under OM conditions with H_2_O_2_ treatment, SIRT1‐overexpression MSCs expressed higher ALP activity than vector‐transfected MSCs. Moreover, overexpression of SIRT1 could restore the expression of ALP activity in MSCs cultured in OM with H_2_O_2_ treatment to levels seen when cultured in OM alone without H_2_O_2_ (Fig. 4D). These findings demonstrate that reconstitution of SIRT1 has a critical and sufficient role in reversing H_2_O_2_‐mediated MSC adipogenesis/osteogenesis lineage switching.

### SIRT1 agonism *via* resveratrol promotes osteogenesis over adipogenesis under conditions of oxidative stress, and deacetylation of FOXO3a with subsequent RUNX2 transactivation is involved

**Figure 5:**
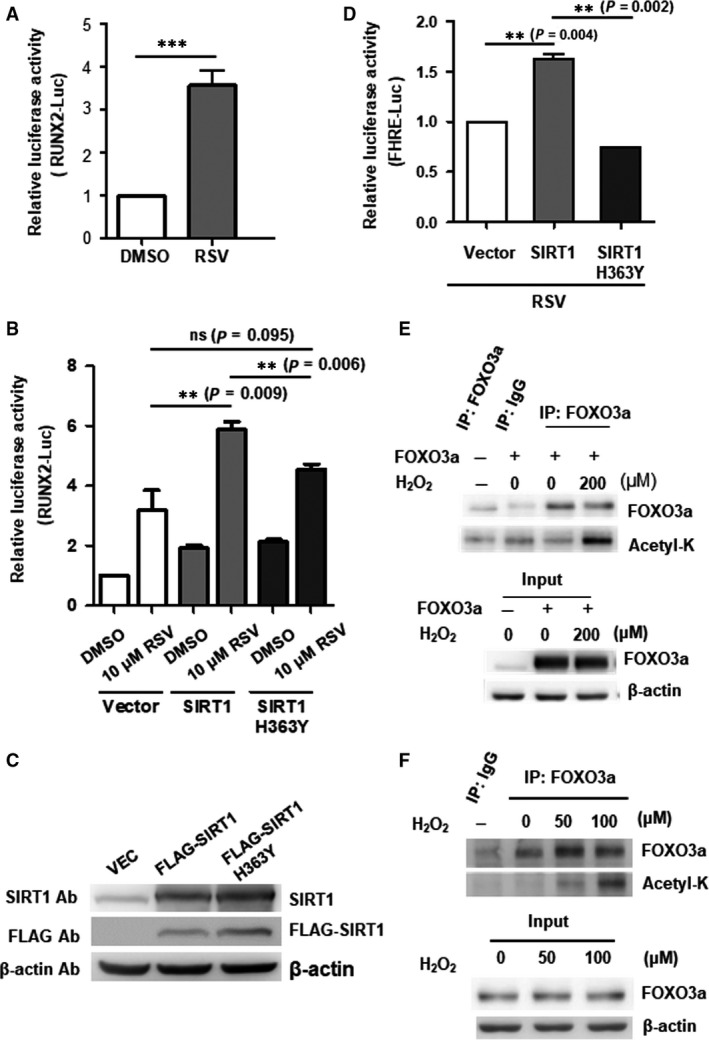
SIRT1 agonism under oxidative stress can regulate the transcriptional activity of RUNX2 by deacetylating FOXO3a. (**A**) RUNX2 promoter activity in 293T cells after treatment without or with resveratrol (RSV). Luciferase activity is shown as fold‐change to control (DMSO) and as mean ± S.D. (**B**) Role of SIRT1 on RUNX2 promoter activity was assessed by transient cotransfection of the RUNX2‐Luc promoter reporter, along with either empty vector (pECE; vector), wild‐type SIRT1 overexpression vector (SIRT1) or mutant SIRT1 overexpression vector (H363Y) in 293T cells, with addition of resveratrol (RSV, 10 μM) 24 hrs after transfection. Luciferase activity was assessed and indicated as fold‐change over control (DMSO) and as mean ± S.D. (**C**) Protein expression of wild‐type and mutant SIRT1 was verified by Western blotting. (**D**) Role of SIRT1 on the Foxo3a‐binding element, forkhead response element (FHRE), was assessed by transient cotransfection of FHRE‐luc reporter, along with empty vector, Sirt1 or mutant SIRT1 in 293T cells with addition of RSV 24 hrs after transfection. Luciferase activity was assessed and indicated as fold‐change over control (DMSO) and as mean ± S.D. (**E**,** F**) Effect of H_2_O_2_ on acetylation status of Foxo3a as assessed by immunoprecipitation (IP) and immunoblotting. (**E**) 293T cells were transfected with FOXO3a expression plasmid (FOXO3a) and then treated with H_2_O_2_ as indicated_._ FOXO3a was immunoprecipitated (IP), and immunoblotting with antibodies to acetylated lysine (Acetyl‐K) was performed to detect acetylation of immunoprecipitated FOXO3a. The amounts of Foxo3a and the loading control β‐actin are shown in the input. (**F**) Immunoprecipitation of endogenous FOXO3a from H_2_O_2_‐treated C3H cells with subsequent immunoblotting using the indicated antibodies for detection of acetylated‐FOXO3a. The input lane shows the expression of FOXO3a and β‐actin. ***, *P* < 0.001, **, *P* < 0.01; ns, non‐significant.

To investigate the mechanism by which SIRT1 promotes osteogenesis over adipogenesis under oxidative stress, we first assessed whether the transcriptional activity of RUNX2, the master osteogenesis transcription factor, was affected. Using the SIRT1 agonist resveratrol (RSV), we found that RUNX2 promoter activity can be enhanced (Fig. 5A). To ascertain whether the deacetylase activity of SIRT1 was involved in this process, we performed overexpression of either wild‐type SIRT1 or SIRT1 H363Y, a deacetylase‐inactive point mutant of SIRT (mutant SIRT1) 22, in C3H MSCs, and verified these protein expressions in Figure 5C. Addition of RSV demonstrated that in C3H with wild‐type SIRT1 overexpression, RUNX2 promoter activity was significantly increased over C3H MSCs without SIRT1 overexpression (Fig. 5B). However, addition of RSV to C3H MSCs with overexpression of mutant SIRT1 did not demonstrate increased RUNX2 promoter activity compared to vector‐transfected C3H MSCs (Fig. 5B), indicating that the deacetylase activity of SIRT1 was necessary for transcriptional control of RUNX2.

We previously demonstrated that SIRT1‐mediated anabolic osteogenic effects in MSCs required FOXO3a, an important partner of SIRT1, through binding of a SIRT1/FOXO3a complex to a novel FOXO response element (FRE) on the proximal promoter of RUNX2, which then result in up‐regulation of RUNX2 expression 20. To assess whether the deacetylase activity of SIRT1 was involved in FOXO3a transcriptional activity, we transfected either wild‐type or mutant SIRT1 along with a promoter–luciferase reporter containing FOXO3a binding site (FHRE). Under RSV treatment, we found that overexpression of wild‐type SIRT1 significantly induced FHRE promoter activity compared to mutant SIRT1 (Fig. 5D), suggesting that deacetylation of FOXO3a by SIRT1 and further SIRT1 agonism can enhance expression of FOXO3a target genes. In addition to being an important cofactor of SIRT1, FOXO3a is a critical mediator of antioxidant activities 22 and is acetylated under oxidative stress. We found that exogenous H_2_O_2_ increased the acetylation status of FOXO3a in 293T cells, as demonstrated by immunoprecipitation of FOXO3a with immunoblotting analyses using antibodies to acetylated lysine (Fig. 5E). Moreover, ROS can induce acetylation of endogenous FOXO3a proteins in C3H MSCs as well (Fig. 5F). Overall, these findings demonstrate that deacetylation of FOXO3a by SIRT1 is required for the transcriptional transactivation of RUNX2 under conditions of oxidative stress.

## Discussion

**Figure 6:**
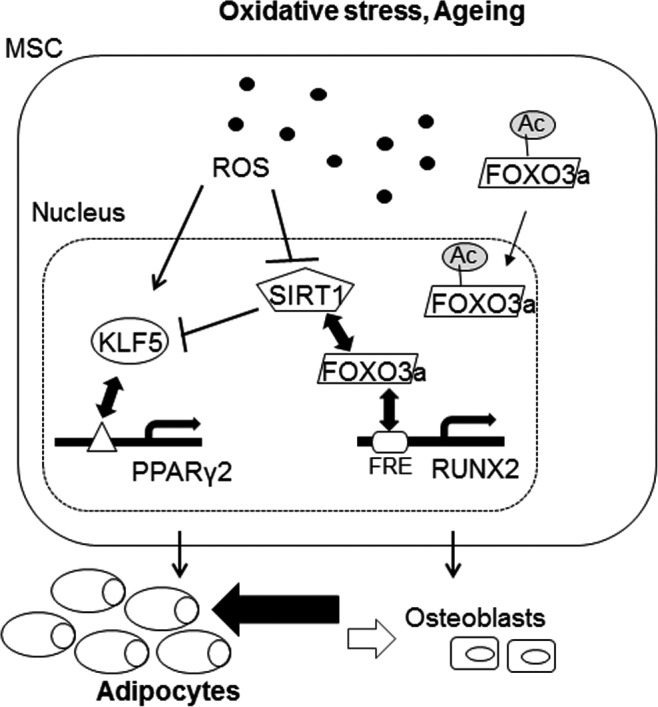
SIRT1 is critically involved in reactive oxygen species (ROS)‐mediated modulation of mesenchymal stem cell (MSC) adipogenesis/osteogenesis transcriptional machinery. SIRT1 up‐regulation of RUNX2 expression is mediated through deacetylation of FOXO3a, while its down‐regulation of PPARγ expression is mediated through inhibition of KLF5 expression levels, an upstream transcription factor of PPARγ. Excessive ROS generated by oxidative stress and/or ageing leads to suppression of SIRT1 protein expression, which results in increased PPARγ2 expression and decreased RUNX2 expression. Moreover, ROS itself also enhances KLF5 expression, reinforcing adipogenesis. Consequently, under conditions of excessive ROS, MSC differentiation capacity is biased towards adipocytes away from osteoblasts.

ROS formed due to oxidative stress has been linked to enhanced adipocyte formation with impaired bone generative potential in MSCs, and this appears to be a potentially aggravating factor in postmenopausal and/or senescence‐correlated osteoporosis 3, 15. However, the molecular basis of ROS modulation on MSC lineage commitment is surprisingly unclear. In this report, we demonstrate the critical link of SIRT1 to ROS‐mediated lineage commitment of MSCs. As stem cells, MSCs possess multilineage capacity that can be modulated by environmental cues, including ROS generated from oxidative stress and senescence. Studies have shown that ROS enhance MSC adipogenesis with involvement of PPARγ2 and C/EBPβ 16; conversely, other reports demonstrate that ROS suppress osteogenic commitment through canonical Wnt/β‐catenin signalling 15, 36 or decreased c‐MAF expression, an ROS‐sensitive cofactor of RUNX2 23, 33. Clearly, while the mechanisms involved in ROS modulation of either of these two lineages have been independently investigated, the molecular events which simultaneously coordinate both lineages remain largely unexplored. Our data demonstrate that ROS simultaneously affect both transcriptional programmes of MSC adipogenesis and osteogenesis, and that SIRT1 has a pivotal role in such oxidative stress‐mediated MSC adipogenesis/osteogenesis lineage switching (Fig. 6). Down‐regulation of SIRT1

Stem cell lineage commitment is mainly controlled by master lineage transcription factors, and we found that ROS up‐regulated the adipogenic transcriptional programme—KLF5, C/EBPβ, PPARγ2 and LEPTIN—while simultaneously down‐regulating the osteogenic transcriptional programme—RUNX2, c‐MAF and COL1A—in MSCs. SIRT1 has been reported to repress PPARγ in differentiated white adipocytes 19, and we previously demonstrated that SIRT1 promotes RUNX2 expression through FOXO3a 20. Our findings in this report demonstrate that SIRT1 is a pivotal player in the transcriptional control of MSC adipogenesis/osteogenesis under oxidative stress. SIRT1 was first discovered to be a histone deacetylase, and later found to play critical roles in metabolism and organismal ageing 18, 37. In MSC biology, SIRT1 has been shown to promote long‐term growth and resistance to senescence 38. We found that SIRT1 expression in MSCs was decreased with high levels of H_2_O_2_ (Figs. 3A), and modulation of SIRT1 levels by knockdown (Fig. 3C and D) or overexpression (Fig. 4C and D) can shift the balance of MSC osteogenesis/adipogenesis under oxidative stress. These findings not only demonstrate strong connection between SIRT1 and ROS‐regulated transcriptional machinery of MSC lineage commitment, but also highlight this molecule as an anabolic target for ROS‐induced osteogenic decline.

Intriguingly, we noticed that knockdown of SIRT1 strongly enhanced MSC adipogenesis, but inhibited osteogenesis to a lesser degree (Figs 3D, E, and 5B). It has been well documented that adipogenesis is easily induced in MSCs by many conditions including senescence, oxidative stress/ROS and even ROS intrinsic to the adipogenesis process itself 17, 23, 39, 40. Such a propensity of MSCs to more readily undergo adipogenesis may likely explain why ROS‐induced SIRT1 antagonism or depletion easily induces expression of adipocyte‐specific genes and adipogenesis, but does not lead to an equivalent level of osteogenic‐specific genes or osteogenesis down‐regulation at the same time. Further research is urgently needed to unravel the molecular machinery and environmental influences responsible for this apparently intrinsic imbalance in the adipogenic/osteogenic potential of MSCs.

Cellular response to oxidative stress is coordinated, and FOXO3a is one of the most important response molecules in this process. This transcriptional factor is also an evolutionarily preserved target of SIRT1 as well as a critical molecule by which major SIRT1 functions are exerted 41. We previously demonstrated that the osteogenic actions of SIRT1 required its interaction with FOXO3a, which then bind to the RUNX2 promoter to activate MSC osteogenic commitment 20. In this report, we found that under oxidative stress, FOXO3a is hyperacetylated and requires SIRT1 deacetylation to restore RUNX2 transactivation for MSC osteogenic differentiation to proceed (Fig. 5). In addition to our laboratory's and others’ work demonstrating how ROS and cellular senescence decrease the expression of c‐MAF, a cofactor of RUNX2 17, 33, this study adds to the molecular understanding on how ROS and oxidative stress adversely impact on osteogenesis. Collectively, these reports highlight the importance of decreasing oxidative stress and ROS—levels which correlate strongly with age—for MSC osteogenesis to occur.

In summary, we found that H_2_O_2_, an ROS, induced MSC lineage commitment towards adipogenesis and away from osteogenesis simultaneously through modulation of master lineage transcription factors and cofactors: PPARγ2, CEBPβ and KLF5 for adipogenesis, and RUNX2 and c‐MAF for osteogenesis. ROS modulation of MSC adipogenic/osteogenic lineage commitment also involves SIRT1, and reconstitution of SIRT1—or agonism with RSV—can reverse the effects of decreased osteogenesis through SIRT1 deacetylation of FOXO3a, itself an ROS‐sensitive molecule, which then allows for FOXO3a binding and transactivation of RUNX2. Taken together, our data implicate SIRT1 as playing a vital role in ROS‐directed lineage commitment of MSCs by modulating two lineages simultaneously. Ultimately, our findings help demonstrate the molecular mechanism involved in ROS‐regulated lineage commitment of MSCs and will facilitate discovery of druggable anabolic targets for use towards ROS/age‐induced osteoporosis.

## Conflicts of interest

All authors state that they have no conflict of interests.
